# Characteristics of patients who use yoga for pain management in Indian yoga and naturopathy settings: a retrospective review of electronic medical records

**DOI:** 10.3389/fpain.2023.1185280

**Published:** 2023-06-15

**Authors:** Pradeep M. K. Nair, Sucheta Kriplani, Prakash Babu Kodali, Ayush Maheshwari, Kinjal Dilipsinh Bhalavat, Deepika Singh, Sanjeev Saini, Dinesh Yadav, Jyoti Keswani, Karishma Silwal, Hemanshu Sharma, Gulab Rai Tewani

**Affiliations:** ^1^ Department of Research, Sant Hirdaram Medical College of Naturopathy and Yogic Sciences for Women, Bhopal, India; ^2^ Department of Yoga and Naturopathy, Sant Hirdaram Yoga and Nature Cure Hospital, Bhopal, India; ^3^ Department of Public Health and Community Medicine, Central University of Kerala, Kasaragod, India; ^4^ Department of Physiology, Morarji Desai Institute of Naturopathy and Yoga Sciences, Vadodara, India; ^5^ Department of Yoga, Sant Hirdaram Medical College of Naturopathy and Yogic Sciences for Women, Bhopal, India; ^6^ Department of Community Medicine, Sant Hirdaram Medical College of Naturopathy and Yogic Sciences for Women, Bhopal, India

**Keywords:** yoga, pain, integrative medicine, palliative care, patient demographics, yoga adherence

## Abstract

**Objectives:**

The aim of this study is to identify the characteristics of patients who underwent yoga therapy for pain in yoga and naturopathy clinical settings in India.

**Methods:**

Electronic medical records of patients who received yoga therapy for pain in three inpatient yoga and naturopathy hospitals were reviewed retrospectively from January 2021 to September 2022. Demographic characteristics and details on pain condition, socioeconomic status, comorbidities, ancillary therapies received, and insurance status were collected. In addition, we prospectively collected data on adherence to yoga practice through a telephonic interview.

**Results:**

A total of 984 patients were identified from a pool of 3,164 patients who received yoga therapy for pain for an average of 9.48 (1.13) days. Patients aged between 8 and 80 underwent therapy for varying pain conditions and diseases that include pain in the extremities, pain due to infection, trauma, degenerative diseases, autoimmune diseases, and spine and neurological diseases. The majority of the patients were females (66.3%), from middle class families (74.8%), and who did not have any insurance coverage (93.8%). Most of the patients were under naturopathy treatment (99.8%), followed by ayurveda (56%), and physiotherapy (49.3%), along with yoga therapy. All patients reported a significant reduction in pain post-integrated yoga therapy (*p* < 0.001). Adherence to yoga was significantly associated with underlying pain conditions, the presence of comorbidities, the types of therapies used, and socioeconomic status (*p* < 0.001).

**Conclusion:**

This study highlights the real-time application of yoga in pain management in Indian yoga and naturopathy settings, as well as implications for future research.

## 1. Introduction

The popularity of yoga across the globe has grown over the years. This surge in the acceptability of yoga has made it one of the most prominently used complementary medicine therapies for various medical conditions (1). Evidence also supports the use of yoga for various medical conditions. However, little is known about the characteristics and socioeconomic background of patients who use yoga as a treatment modality, especially those of the Indian population. A recent cross-sectional study from India reported a prevalence rate of 11.8% for yogic practice among Indians(2). However, the primary focus of this survey is to understand the usefulness of yoga in diabetes mellitus. Barring this survey, no other reports are available on the characteristics of patients from India who use yoga for different medical conditions.

Understanding the characteristics, socioeconomic background, and duration of illness of patients who use yoga as a therapy will provide useful insights that may aid in devising public health policies. While the beneficial effect of yoga in managing psychological and metabolic conditions is well known, there is a growing interest in using yoga for pain management as well (3, 4). However, a recent review indicates that the majority of the studies reporting the efficacy of yoga in pain are related to backache, migraine, and fibromyalgia (3). Presently, there is no literature available regarding the use of yoga in pain conditions based on socioeconomic status, clinical condition, associated comorbidities, or the concomitant therapies used alongside yoga therapy.

In India, yoga and naturopathy are regarded among the official indigenous systems of medicine under the Ministry of Ayurveda, Yoga and Naturopathy, Unani, Siddha, Sowa-Rigpa, and Homoeopathy (AYUSH), Government of India (5). In Indian yoga and naturopathy hospitals, yoga therapy prescription is made by yoga and naturopathy physicians who have a Bachelors' degree in Yoga and Naturopathy (BNYS—Bachelor of Naturopathy and Yogic Sciences) and an “A” class medical registration in their respective state medical councils (6). A typical yoga and naturopathy inpatient setting offers therapies like diet therapy, heliotherapy, acupressure, acupuncture, hydrotherapy, massage therapy, and yoga therapy (6).

There are approximately 5,000 yoga and naturopathy hospitals in India (7) that use yoga therapy as an integral part of their treatment plan for all conditions, including pain. However, little is known about the pattern of clinical practice in these hospitals. Mapping the clinical patterns of yoga usage among patients will provide information on the current prescription practices of yoga, factors associated with its adherence, commonly used conditions, and so on, which will help in policy formulation and research. Therefore, in this study, we focus on the use of yoga for pain management and aim to provide information on the characteristics of patients who sought yoga as a therapy for their pain conditions in three yoga- and naturopathy-based inpatient hospitals from three different parts of India.

## 2. Methodology

### 2.1. Study design

This study used a cross-sectional study design. The medical records of patients treated with yoga alone or in combination with other complementary medicine therapies for pain were retrospectively reviewed between January 2021 and September 2022 at three in-patient yoga and naturopathy settings in the north, south, and central parts of India . These hospitals were purposefully selected as they had an average bed occupancy rate of over 60% throughout the year.

### 2.2. Data sources

The electronic medical records were reviewed to collect data on the patients' sociodemographic characteristics such as age, gender, economic status [computed using the Kuppuswamy scale (8)], insurance coverage, pain condition for which the patient underwent yoga therapy (refer Supplementary File), duration of the pain condition, additional treatments received along with yoga therapy, and visual analog scores for pain. Further, we prospectively collected data on adherence to yoga practice through a telephonic interview. An individual was considered adhering to practice if they reported that they continued to practice yoga at least once a week post discharge.

### 2.3. Study criteria

All patients who were treated as inpatients for pain (see Supplementary File) exclusively with yoga or yoga integrated with other complementary medicine therapies (CAM) such as naturopathy, ayurveda, and physiotherapy at the three study settings were included in the study. Patients who were treated with other CAM modalities, excluding yoga, outpatients, patients whose electronic medical records were incomplete, and those who were treated for conditions other than pain were excluded from the study.

### 2.4. Data synthesis

We analyzed the data of 984 patients who were treated for pain using yoga or yoga with other complementary medicine therapies in the three selected study sites. Prior to analysis, we cleaned the data to address issues relating to any missing data, data entry errors, and inconsistencies. The missing data were found to be minimal (*n* = 6) and were observed only under the category of variable age. We employed the item mean substitution method (9) to replace these missing data. The variable age was classified into three categories using class intervals. The categories in the variables such as non-communicable disease (NCD) status, socioeconomic status, and use of complementary/multiple therapies were collapsed into fewer categories to facilitate the analysis. Frequency tables were constructed to study the characteristics of patients and the characteristics of the therapy received.

The pain scores captured using the Visual Analog Scale (VAS) were descriptively analyzed using mean and standard deviation. Normality of the pain scores was assessed using the skewness of the scores and Q–Q plots. The pretherapy pain scores had a near normal distribution with a skewness of 0.239. We compared the differences in pre- and post-therapy VAS pain scores across the patient characteristics using linear mixed models (LMMs). The effect size was calculated using *Glass delta* (10) to measure the magnitude and direction of difference between pain scores before and after treatment with yoga or yoga integrated with other complementary medicine therapies.

The patients were followed up to determine their levels of adherence to yogic practice after discharge. A binary logistic regression analysis was conducted to assess the factors associated with adherence to yoga among the participants. The Hosmer–Lemeshow test was conducted to identify the model fit. Adjusted odds ratios (AORs) and 95% CI for the AORs were computed.

### 2.5. Ethical considerations

The study was approved by the Institutional Ethics Committee of Sant Hirdaram Medical College of Naturopathy and Yogic Sciences for Women (F.No:12/SHMCNYS-IEC/P27/2022-23), Prakriti Shakti Clinic of Natural Medicine (CGH-PS/IRB/2022/2), and Morarji Desai Institute of Naturopathy and Yogic Sciences (MDINYS/IEC/2022/P7). All participants signed an informed consent (obtained during their in-patient stay for using their de-identified data for research purposes) form to participate in the study.

## 3. Results

### 3.1. Baseline characteristics of the patients

The three study sites in total had 3,764 inpatient admissions, with the average stay of patients being 9.48 ± 1.13 days. Of these 3,764, 26.14% (*n* = 984) were found to have undergone a yoga therapy session for a pain condition. These 984 patients accounted for the study sample. The study participants included 66.3% (*n* = 652) females and were primarily in the age group of 34–58 years (55.1%, *n* = 542). All participants enrolled reported suffering from some form of pain, with pain due to spine and neurological conditions being the most prominent (36.2%). Most of the participants (93.8%) who sought yoga therapy were not covered by any insurance policies (see Table 1).

**Table 1:** Characteristics of the study participants (*n* = 984).

Variable	Frequency	Percentage
Sex of the participant		
Male	332	33.7
Female	652	66.3
Age group		
Up to 33 years	63	6.4
34–58 years	542	55.1
59 years and above	379	38.5
Socioeconomic status		
Lower	98	10.0
Middle	736	74.8
Upper	150	15.2
NCD status		
Nil	162	16.5
Cardiovascular disease	185	18.8
Gastrointestinal disorders	140	14.2
Endocrine and metabolic disorders	283	28.8
Reproductive tract issues	31	3.2
Respiratory diseases	35	3.6
Others	148	15.0
NCD multimorbidity		
No NCD	162	16.5
Single NCD	371	37.7
Multimorbidity	451	45.8
Pain condition		
Lower extremity pain	238	24.2
Upper extremity pain	38	3.9
Pain in upper and lower extremities	49	5.0
Infection, injury, and surgery	13	1.3
Degenerative and autoimmune diseases	223	22.7
Spine and neurological diseases	356	36.2
Others	67	6.8
Insurance coverage		
Covered under insurance	61	6.2
Not covered	923	93.8
Type of therapy		
Naturopathy	982	99.80
Physiotherapy	485	49.30
Ayurveda	551	56.00
Use of multiple therapies		
Yoga and naturopathy only	180	18.30
Mix of yoga, naturopathy, and other therapies	804	81.70

Others, ayurveda, and physiotherapy.

### 3.2. Use of concomitant therapies along with yoga

**Figure 1:**
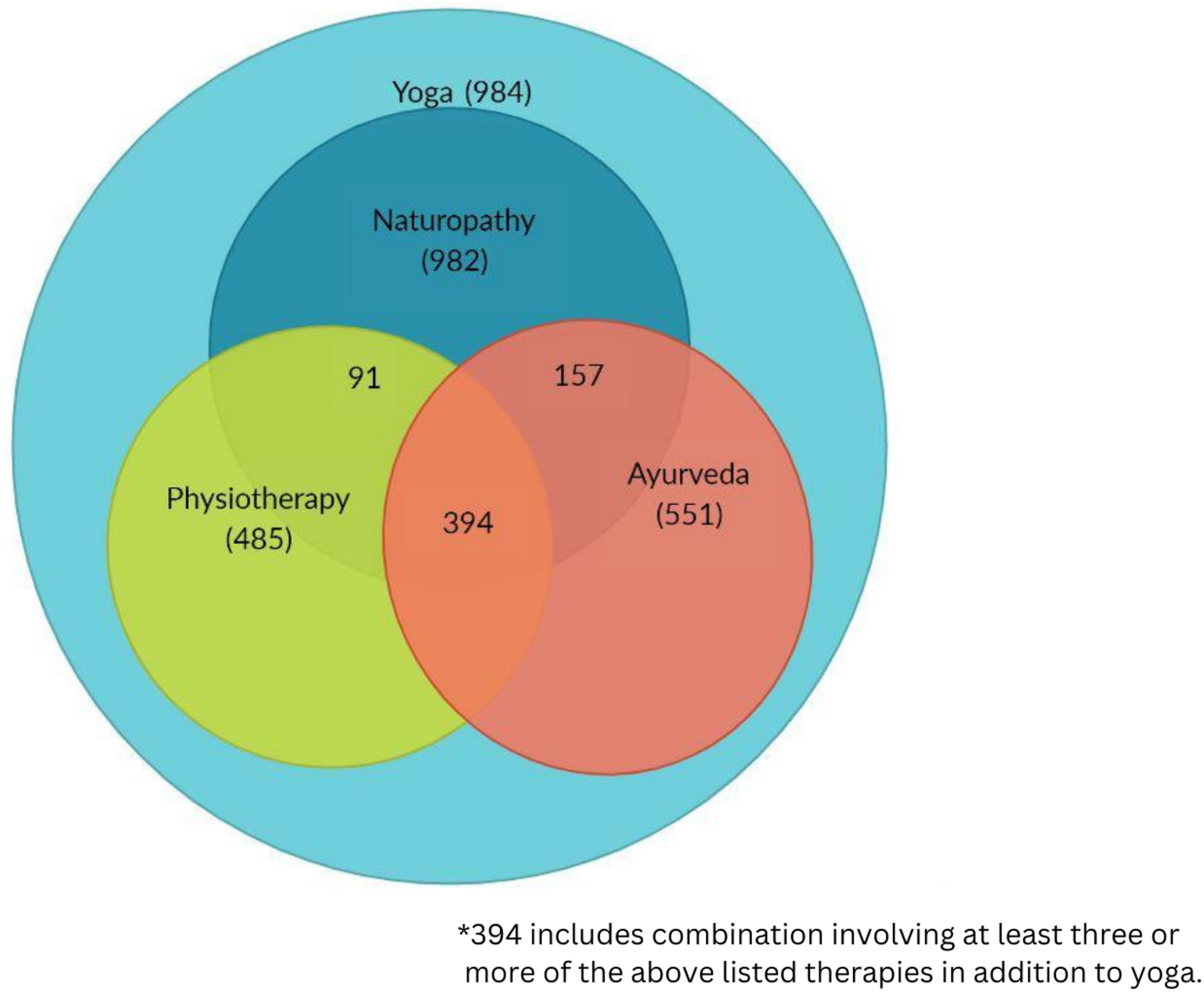
Schematic representation outlining the use of multiple therapies among the study participants.

We noted that most of the participants received more than one type of therapy in addition to their yoga therapy. Among the participants who were receiving naturopathy, a substantial proportion also received ayurveda and physiotherapy (see Figure 1). Differentials in the use of various forms of therapy across demographic and health status were noted among the study participants (see Table 2).

**Table 2:** Characteristics of the study participants in terms of the use of naturopathy and other complementary therapies along with yoga (*n* = 984).

Variables	Naturopathy (*n* = 982) (%)	Physiotherapy (*n* = 485) (%)	Ayurveda (*n* = 551) (%)
Sex of the participant			
Male	100.00	42.80	51.80
Female	99.70	52.60	58.10
Age group			
Up to 33 years	100.00	50.80	60.30
34–58 years	99.60	45.40	51.70
59 years and above	100.00	54.60	61.50
NCD status			
Nil	99.40	21.00	44.40
Cardiovascular disease	100.00	43.80	52.40
Gastrointestinal disorders	100.00	55.70	57.90
Endocrine and metabolic disorders	99.60	53.70	57.20
Reproductive tract issues	100.00	67.70	71.00
Respiratory diseases	100.00	57.10	65.70
Others	100.00	66.90	63.50
NCD multimorbidity			
No NCD	99.40	21.00	44.40
Single NCD	99.70	29.60	41.00
Multimorbidity	100.00	75.60	72.50
Pain condition			
Lower extremity pain	100.00	75.60	70.20
Upper extremity pain	97.40	52.60	42.10
Pain in upper and lower extremities	100.00	95.90	91.80
Infection, injury, and surgery	100.00	84.60	84.60
Degenerative and autoimmune diseases	99.60	20.30	40.80
Spine and neurological diseases	100.00	43.30	53.10
Others	100.00	40.30	47.80
Insurance coverage			
Not covered	99.80	51.00	56.90
Covered under insurance	100.00	23.00	42.60

NCD, non-communicable diseases.

### 3.3. Impact of yoga in pain management

The VAS pain scores were captured prior to the therapy and after the therapy among study participants during their inpatient stay. The LMMs were used to assess the changes in VAS scores before and after therapy across the patient characteristics. The findings indicate that yoga therapy resulted in a significant pain reduction (see Table 3).

**Table 3:** Changes in the VAS pain score pre- and post therapy among the study participants (*n* = 984).

Patient characteristics	VAS-pain pre	Vas-pain post	Effect size	*p*-Value*
Sex				
Male	5.44 (±2.49)	1.54 (±1.32)	−1.57	
Female	5.95 (±2.40)	1.58 (±1.42)	−1.81	0.005
Pain condition				
Lower extremity pain	6.83 (±2.46)	1.19 (±1.25)	−2.29	
Upper extremity pain	4.84 (±2.40)	1.92 (±1.75)	−1.22	
Pain in upper and lower extremities	8.20 (±2.50)	1.41 (±2.07)	−2.72	
Infection, injury, and surgery	7.08 (±2.02)	1.00 (±0.58)	−3.01	
Degenerative and autoimmune diseases	4.61 (±1.84)	1.95 (±1.40)	−1.45	
Spine and neurological diseases	5.51 (±2.38)	1.62 (±1.31)	−1.63	
Others	5.89 (±2.39)	1.26 (±1.02)	−1.94	0.000
NCD status				
No NCD	4.41 (±1.52)	1.79 (±1.20)	−1.72	
Single NCD	4.96 (±2.13)	1.74 (±1.33)	−1.51	
NCD multimorbidity	6.95 (±2.44)	1.33 (±1.46)	−2.30	0.000
Therapy				
Yoga and naturopathy only	4.29 (±1.63)	1.91 (±1.35)	−1.46	
Mix of yoga, naturopathy, and other therapies	6.10 (±2.47)	1.48 (±1.38)	−1.87	0.000
Age group				
Up to 33 years	6.10 (±2.62)	1.33 (±1.15)	−1.82	
34–58 years	5.52 (±2.38)	1.60 (±1.42)	−1.65	
59 years and above	6.07 (±2.47)	1.55 (±1.36)	−1.83	0.047
Insurance coverage				
Not covered	5.87 (±2.48)	1.54 (±1.39)	−1.75	
Covered under insurance	4.42 (±1.14)	1.89 (±1.08)	−2.22	0.005

* Linear mixed models were used to compare the pre- and post differences in pain scores across patient characteristics.

### 3.4. Factors associated with adherence to yoga therapy among pain patients

It was found that 75.3% (*n* = 741) reported continuing yoga after being discharged from the facility. Specifically, 39.8% (*n* = 392) practiced yoga every day of the week, 10.7% (*n* = 105) practiced yoga thrice a week, and 24.8% (*n* = 244) practiced yoga up to twice a week. Patients who were suffering from lower extremity pain (AOR = 2.05; 95% CI = 1.05–3.99), those who had NCD multimorbidity (AOR = 3.93; 95% CI = 2.47–6.25), and those who belonged to the middle (AOR = 2.27; 95% CI = 1.44–3.58) and upper (AOR = 2.02; 95% *CI* = 1.06–3.85) socioeconomic strata had greater odds of adhering to yoga therapy post discharge (see Table 4).

**Table 4:** Factors associated with adherence to yoga among the study participants (*n* = 984).

Patient characteristics	Adhering to Yoga (%)		AOR (95% CI)	*p*-Value
	No (*n* = 243)	Yes (*n* = 741)		
Sex				
Male (ref)	37.00	32.70		
Female	63.00	67.30	1.11 (0.80–1.54)	0.549
Pain condition				
Others (ref)	8.60	6.20		
Lower extremity pain	13.60	27.70	2.05 (1.05–3.99)	0.036
Upper extremity pain	4.10	3.80	1.07 (0.41–2.77)	0.890
Pain in upper and lower extremities	1.60	6.10	2.89 (0.89–9.39)	0.077
Infection, injury, and surgery	0.80	1.10	2.20 (0.43–11.29)	0.346
Degenerative and autoimmune diseases	33.70	19.00	0.94 (0.51–1.76)	0.854
Spine and neurological diseases	37.40	35.80	1.36 (0.75–2.47)	0.319
NCD status				
No NCD (ref)	27.60	12.80		
Single NCD	49.80	33.70	1.38 (0.93–2.05)	0.112
NCD Multimorbidity	22.60	53.40	3.93 (2.47–6.25)	0.000
Therapy received				
Yoga and naturopathy only (ref)	27.20	15.40		
Mix of yoga, naturopathy, and other therapies	72.80	84.60	1.29 (0.89–1.88)	0.185
Age group				
Up to 33 years (ref)	6.60	6.30		
34–58 years	54.30	55.30	1.03 (0.54–1.97)	0.931
59 years and above	39.10	38.30	0.80 (0.41–1.55)	0.502
Socioeconomic status				
Lower (ref)	19.30	6.90		
Middle	70.80	76.10	2.27 (1.44–3.58)	0.000
Upper	9.90	17.00	2.02 (1.06–3.85)	0.033
Insurance coverage				
Not Covered (ref)	24.80	75.20		
Covered under Insurance	23.30	77.00	1.81 (0.95–3.45)	0.069

Dependent variable: adhering to yoga: no (ref); yes. Ref: reference category; AOR, adjusted odds ratio; CI, confidence interval.

## 4. Discussion

The present study is the first of its kind that showcases the characteristics of patients who use yoga for treating their pain conditions, from three yoga and naturopathy clinical settings in India. Our findings indicate that females and patients over the age of 34 are more likely to use yoga for pain management. This may be largely attributed to the gender predisposition of pain, wherein females suffer more from pain than their male counterparts (10). Similarly, the increased prevalence of the use of yoga for pain management among older adults can be attributed to the increased prevalence of chronic pain among older adults compared with their younger counterparts (10).

Our data suggest socioeconomic status to be a significant predictor for the use of yoga therapy for pain. Most of the patients who underwent yoga therapy for pain were from either the middle class or the upper class. This indicates the need for formulating policies that enhance the availability of yoga among the lower socioeconomic strata of society, as a lower socioeconomic status or financial instability is reported to increase the vulnerability to pain (11, 12).

Barring 16% of the patients, nearly one-third of the patients who underwent yoga therapy for pain had at least one NCD. Endocrine and metabolic disorders accounted for 50% of NCDs among the patients, and more than 46% of them had more than one NCD. This indicates the coexistence of NCDs and pain conditions. The need for providing palliative care among NCD patients is seldom suggested and is often considered as a secondary goal (13, 14). Our study population presenting with an NCD comorbidity along with pain indicates the need for introducing palliative care among NCD patients. The potential for integration of yoga as part of palliative care for patients suffering from NCDs should be further explored.

An earlier review reported that, most of the studies describing the efficacy of yoga in pain were related to chronic back pain (3). Underreporting, limited expertise, lack of experience, and/or lack of awareness on the importance of research among complementary and alternative medicine practitioners (15) may be a primary reason for the research reports to be confined to backache. Our case chart review suggests that yoga is being used for an array of pain conditions that are not limited to backache. This indicates that research on the area of yoga and pain should be expanded beyond backache by including patients from yoga- and naturopathy-based hospitals. Further, adequate exposure to research and journal communications are warranted for yoga and naturopathy physicians to unveil the magnitude of yoga in pain management.

In this study, it was found that a majority of the patients who availed yoga therapy for pain had no insurance coverage, reflecting out-of-pocket spending for accessing yoga services. This is perplexing, because, on the one hand, yoga is being immensely popularized by policymakers, and on the other hand, there exists shortcomings in policies that limit its accessibility. Numerous commentaries and reviews have opined that the non-availability of insurance coverage for yoga may restrict its use only among the upper strata of the society (3, 16, 17). Moreover, the limited focus of health insurance for yoga and complementary medical systems also comes in the way of the integration of yoga services with universal healthcare.

In this study, it was found that almost all patients who used yoga for pain management have also used another complementary and alternative therapy (CAM). This pattern was consistent across the three study settings, and naturopathy was reported as the mostly used therapy, followed by ayurveda. This may be attributed to prescription practice followed at the respective clinical settings. Further, the patients may have co-opted to undergo these therapies because of their comorbid conditions, as an integrated approach is reported to reduce the burden of NCDs (18–20). The report on the use of CAM therapies in our patients reiterates the integrative potential of yoga therapy with other therapies.

All patients reported a significant reduction in their pain irrespective of their age, clinical condition, NCD status, concomitant therapies used, and insurance status. This observation is consistent with that of other experimental studies reporting yoga to alleviate pain in various conditions (21–24). We also prospectively explored the adherence/continuity to yogic practice among the patients and found that 75% of them continued the practice after their initial exposure in the clinical setting. Adherence to yogic practice was associated with the pain condition of the patient, the presence of NCD, and the socioeconomic status. Furthermore, we did not gather any information about the time between the patient's discharge and the time of data collection. This could also play a part in adhering to yoga, and the lack of information on this could be regarded as a limitation of this study.

Patients with multiple NCD conditions were found to adhere more to yogic practice compared with those with a single NCD or no NCD. NCD multimorbidity is associated with significant burden and stress. Yoga integrated with naturopathy is recommended to reduce the burden and associated complications of NCD (18). An earlier community-based observational study from India also reported increased utilization of yoga therapy along with naturopathy among NCD patients (25). The heightened adherence to yoga among patients with multimorbid NCDs, which was found in the present study, may be viewed as an increasing acceptance of yoga therapy among patients.

Socioeconomic status has emerged as another important factor determining the adherence to yoga after the initial exposure. Confirming the results from earlier studies, our study also found a higher adherence of yoga among patients with a higher socioeconomic status (26, 27). Correlating the non-availability of insurance, reduced use and adherence of yoga among the lower socioeconomic group of patients in this study indicates the need for framing revised policies that improve the accessibility to yoga for this group.

To our knowledge, this is the first study to report the characteristics of patients using yoga for pain management in Indian clinical settings. However, there are notable limitations in the present study. First, barring one variable on adherence to yoga, the rest of our analyses were based on retrospective medical records from three yoga and naturopathy hospitals from India. Therefore, the results of this study have limited generalizability and may not fully reflect the present scenario. All the three clinical settings studied offers yoga and naturopathy as an integrated therapy; therefore, it is difficult to determine whether the patients enrolled for the program exclusively for yoga or to avail both interventions together.

Further, we could not extract any data regarding the type of yoga interventions provided, duration of practice, or the detailed use of other supportive therapies, because a manual extraction of such data was not attempted on the ground that it would be extremely exhaustive to do a manual search owing to the individualistic nature of prescription practice in these settings. This remains one of the significant limitations of this present study. We could not collect any data regarding the characteristics of pain and its impact on patient activities of daily living. Such data may have added more value to the findings of this study.

Our study indicates that yoga was used along with other multiple therapies. These insights suggest that future yoga research should use pragmatic designs that can include the common ancillary treatments with yoga therapy, which can offer meaningful results. Nevertheless, such research should at least enquire about the use of additional therapies with yoga, which can strengthen prescription practices. Large-scale observational studies are warranted in the future that can include the factors influencing patients to avail yoga therapy and its diverse prescription practices.

## 5. Conclusions

Yoga therapy is used by patients for managing diverse pain conditions, of which a majority of patients receive yoga therapy for degenerative and autoimmune diseases. Yoga is commonly used as an integrative therapy along with naturopathy, physiotherapy, and ayurveda in clinical settings. The inferences from the present study will help in designing future pragmatic trials in yoga.

## Data Availability

Publicly available datasets were analyzed in this study. Further inquiries can be directed to the corresponding author/s.

## References

[1] Cramer H Ward L Steel A Lauche R Dobos G Zhang Y. Prevalence, patterns, and predictors of yoga use: results of a U.S. Nationally representative survey. Am J Prev Med. (2016) 50(2):230–5. 10.1016/j.amepre.2015.07.037 26497261

[2] Mishra AS Sk R Hs V Nagarathna R Anand A Bhutani H Knowledge, attitude, and practice of yoga in rural and urban India, KAPY 2017: a nationwide cluster sample survey. Medicines. (2020) 7(2):8. 10.3390/medicines7020008 32033426 PMC7168227

[3] Wren AA Wright MA Carson JW Keefe FJ. Yoga for persistent pain: new findings and directions for an ancient practice. Pain. (2011) 152(3):477. 10.1016/j.pain.2010.11.017 21247696 PMC3040510

[4] Gupta S Gautam S Kumar U Arora T Dada R. Potential role of yoga intervention in the management of chronic non-malignant pain. Evid Based Complement Alternat Med. (2022) 2022:5448671. 10.1155/2022/5448671 35668780 PMC9167073

[5] Ministry of AYUSH. AYUSH Systems. Available at: https://main.ayush.gov.in/ayush-systems/.

[6] Nair PMK Nanda A. Naturopathic medicine in India. Focus Altern Complement Ther. (2014) 19(3):140–7. 10.1111/fct.12125

[7] Tewani GR Sharma H Nathani VV Silwal K Nair PMK. Adverse events reporting at naturopathy clinical settings: importance of accreditation systems in improving quality of care and patient safety. BMJ Open Qual. (2022) 11(4):2088. 10.1136/bmjoq-2022-002088 PMC9764612 36526303

[8] Saleem SM. Modified Kuppuswamy socioeconomic scale updated for the year 2021. Indian J Foransic Community Med. (2021) 8(1):1–3. 10.18231/j.ijfcm.2021.001

[9] Downey RG King CV. Missing data in Likert ratings: a comparison of replacement methods. J Gen Psychol. (1998) 125(2):175–91. 10.1080/00221309809595542 9935342

[10] Glass GV McGaw B Smith ML. Meta-analysis in social research. New Delhi: Sage (1981). Available at: https://www.jameslindlibrary.org/glass-gv-mcgaw-b-smith-ml-1981/

[11] Rios R Zautra AJ. Socioeconomic disparities in pain: the role of economic hardship and daily financial worry. Health Psychol. (2011) 30(1):58. 10.1037/a0022025 21299295 PMC3077089

[12] Ikeda T Sugiyama K Aida J Tsuboya T Watabiki N Kondo K Socioeconomic inequalities in low back pain among older people: the JAGES cross-sectional study. Int J Equity Health. (2019) 18(1):1–11. 10.1186/s12939-019-0918-1 30665404 PMC6341699

[13] Effendy C Silva JFDS Padmawati RS. Identifying palliative care needs of patients with non-communicable diseases in Indonesia using the SPICT tool: a descriptive cross-sectional study. BMC Palliat Care. (2022) 21(1):1–8. 10.1186/s12904-021-00896-y 35073869 PMC8785499

[14] Latour JM Fulbrook P Albarran JW. EfCCNa survey: European intensive care nurses’ attitudes and beliefs towards end-of-life care. Nurs Crit Care. (2009) 14(3):110–21. 10.1111/j.1478-5153.2008.00328.x 19366408

[15] Veziari Y Kumar S Leach M. Barriers to the conduct and application of research among complementary and alternative medicine professions in Australia and New Zealand: a cross-sectional survey. Complement Ther Med. (2021) 60:102752. 10.1016/j.ctim.2021.102752 34126172

[16] Patwardhan AR. Aligning yoga with its evolving role in health care: comments on yoga practice, policy, research. J Prim Care Community Health. (2017) 8(3):176–9. 10.1177/2150131917690092 28166701 PMC5932693

[17] Elgelid S. Insurance reimbursement: what it might mean for the profession of yoga therapy. Int J Yoga Therap. (2001) 11(1):99–102. 10.17761/ijyt.11.1.g87026671347x2v5

[18] Salwa H Nair PMK. Raising burden of non-communicable diseases: importance of integrating yoga and naturopathy at primary care level. J Complement Integr Med. (2020) 18(2):271–8. 10.1515/jcim-2017-0019 32745069

[19] Thomas A Kirschbaum L Crowe BM Van Puymbroeck M Schmid AA. The integration of yoga in physical therapy clinical practice. Complement Ther Med. (2021) 59:102712. 10.1016/j.ctim.2021.102712 33744367

[20] Datey P Hankey A Nagendra HR. Combined ayurveda and yoga practices for newly diagnosed type 2 diabetes mellitus: a controlled trial. Complement Med Res. (2018) 25(1):16–23. 10.1159/000464441 28957795

[21] Tilbrook HE Cox H Hewitt CE Kang’ombe AR Chuang LH Jayakody S Yoga for chronic low back pain: a randomized trial. Ann Intern Med. (2011) 155(9):569–78. 10.7326/0003-4819-155-9-201111010-00003 22041945

[22] Sherman KJ Cherkin DC Wellman RD Cook AJ Hawkes RJ Delaney K A randomized trial comparing yoga, Stretching, and a self-Care book for chronic low back pain. Arch Intern Med. (2011) 171(22):2019–26. 10.1001/archinternmed.2011.524 22025101 PMC3279296

[23] Schmid AA Fruhauf CA Sharp JL Van Puymbroeck M Bair MJ Portz JD. Yoga for people with chronic pain in a community-based setting: a feasibility and pilot RCT. J Evid Based Integr Med. (2019) 24. 10.1177/2515690X19863763 PMC6689911 31394910

[24] Seguin-Fowler R Graham M Ward J Eldridge G Sriram U Fine D. Feasibility of a yoga intervention to decrease pain in older women: a randomized controlled pilot study. BMC Geriatr. (2020) 20(1):1–12. 10.1186/s12877-020-01818-y PMC7552447 33046009

[25] Maheshkumar K Venugopal V Poonguzhali S Mangaiarkarasi N Venkateswaran ST Manavalan N. Trends in the use of yoga and naturopathy based lifestyle clinics for the management of non-communicable diseases (NCDs) in Tamilnadu, South India. Clin Epidemiol Glob Heal. (2020) 8(2):647–51. 10.1016/j.cegh.2019.09.013

[26] Park CL Braun T Siegel T. Who practices yoga? A systematic review of demographic, health-related, and psychosocial factors associated with yoga practice. J Behav Med. (2015) 38(3):460–71. 10.1007/s10865-015-9618-5 25627668

[27] Bishop FL Lewith GT. Who uses CAM? A narrative review of demographic characteristics and health factors associated with CAM use. Evid Based Complement Alternat Med. (2010) 7(1):11–28. 10.1093/ecam/nen023 18955327 PMC2816378

