# Supplementary material for: Characteristics of patients who use yoga for pain management in Indian yoga and naturopathy settings: a retrospective review of electronic medical records
Source: Front Pain Res (Lausanne). 2023 Jun 15;4:1185280. doi: 10.3389/fpain.2023.1185280 (PMC10308043; doi:10.3389/fpain.2023.1185280)
Supplement: Supplementary file 1 [file Table1.docx]

**Supplemental File**

**CATEGORIES OF PAIN**

| **Upper extremity Pain** | Neck pain, Upper back pain, Middle back pain, Interscapular pain, Pain in Bilateral scapular region, Shoulder pain, Bursitis, Frozen shoulder, Elbow pain, Tennis elbow, Wrist pain, Ganglion, Pain in hands/ Hand pain, MND, Finger pain, Trigeminal neuralgia, Eye pain, Ear pain or ear ache, Tooth ache, Keloid, Hand pain with numbness, Thumb pain |
| --- | --- |
| **Lower extremity pain** | Hip joint pain, Pelvic joint pain, Thigh Pain, Sciatica, OA Knee, Knee pain, Arthritis, Calf Muscle Pain, Leg Pain, Leg pain with stiffness, Leg pain with cramps, Feet pain, Corn foot, Heel Pain, Calcaneous spur pain, Flat foot, Plantar fasciitis, Sole pain, Foot drop, Low back pain, Ankle joint pain, Toes pain, Gout |
| **Upper and Lower Extremity Pain** | Multiple joint pain, Whole body pain, Body ache/ Full body pain/ General Body ache, Muscles pain, Muscular pain, Muscular spasm |
| **Neurological Pain** | Head ache, Hemiplegia, Migraine, Parkinson’s Disease, MND |
| **Spine disorders related pain** | Lumbar Spondylosis, Cervical spondylosis, Intervertebral disc prolapse |
| **Post Injuries/ surgery related pain** | Ligament tear, Ankle ligament injury, Post fracture pain, Post piles surgery pain, Hand pain after accident |
| **Pain associated with degenerative Conditions** | Osteoporosis, Osteopenia |
| **Post infection pain** | Chikungunya, Herpes zoster |
| **Hormonal disorders** | Dysmenorrhoea, Hyperuricemia, Menorrhagia related pain, Painful Menstruation |
| **Autoimmune disorders related pain** | Rheumatoid Arthritis, Psoriatic arthritis, Systemic Lupus Erythematosis |
| **Others** | Abdominal pain, Chest Pain, Upper thoracic pain, Gastric pain, Epigastric pain, Gall Bladder (GB) stone, Fistula, Balanitis, Pain in penis, Vaginal pain, Painful Micturition, Throat pain, Stomatitis |

**CATEGORIES OF CO-MORBIDITIES**

| **Psychological Disorders** | Stress, Anxiety, Depression, Memory loss, Anger, Lack of concentration, Claustrophobia |
| --- | --- |
| **Neurological & Cerebrovascular Disorders** | Tremors / numbness in hands, DM Neuropathy, Brain stroke, Myasthenia gravis, Epilepsy, Burning sensation in feet, Cerebral & Cerebellar atrophy, Alzheimer’s disease |
| **Sleep Disorders** | Insomnia, Disturbed/ delayed sleep, Sleep apnea, |
| **Endocrinal Disorders** | Hormonal Imbalance, Hypothyroidism, Hyperthyroidism, Goitrous nodule in thyroid |
| **Eye Disorders** | Eyes burning, Eye dryness, Eye redness, Blur vision, Myopia, Glaucoma, |
| **ENT Disorders** | Hoarseness of voice, Snoring, Vertigo/ Positional vertigo, Itching in ears, Hearing loss, Tinnitus, CSF Rhinorrhoea |
| **Cardiovascular Disorders** | Cardiomegaly, Hypertension, IHD, Palpitation, Varicose veins, CAD, Ventricular Hypertrophy, Sinus tachycardia, Angioplasty, Cardiomegaly, Dyslipidemia |
| **Respiratory Disorders** | Bronchitis/ Allergic Bronchitis, Allergic Rhinitis, Cold, Cough, Sinusitis, Asthma, Hiccups, Nasal Polyps, DNS, Pneumonia |
| **Gastrointestinal Disorders** | Mouth dryness, Nausea, Vomiting, Hyperacidity/ Acidity, Indigestion, Dyspepsis, Loss of appetite, Gas/Flatulence, Bloating/Heaviness, Gastritis, Constipation, IBS, Piles, erosive esophagitis, Fistula, Fissure, Fecal incontinence, Diarrhoea, GERD |
| **Metabolic Disorders** | Obesity/ Central Obesity/Overweight, Insulin Resistance, DM Type II, Dyslipidemia?, Reduction in weight/ Weight loss, Gall bladder stone, Gross Ascitis, Generalized Edema, Local edema/ Puffiness or swelling (Face, Feet, Ankle, Hands), Lipoma, Multiple Myeloma, Carcinoma |
| **Liver Disorders** | Hepatomegaly, Liver Cirrhosis |
| **Female Reproductive Disorders** | Hot flushes, Mood swings, Leucorrhea, Dysmenorhea, Irregular Menstruation, Black menstrual blood, Breast Calcification, Breast cyst, Endometrial polyps, PCOD, Oligomenorrhoea/ Scanty menstruation, Frequent miscarriage, Ovarian cyst, Dry vagina, Infertility, Uterine fibroid, Vaginal itching |
| **Male Reproductive Disorders** | Prostatomegaly/ BPH, , Infertility, Prostate Carcinoma |
| **Kidney Disorders** | CKD, Hyperuricemia, Renal stones, Renal Cortical cyst, |
| **Urinary Disorders** | Burning Micturition, UTI, Dysuria, Urinary incontinence, Oligouria |
| **Skin Disorders** | Fungal infection, Eczema, Hair fall, Alopecia, Dandruff, Skin allergy, Urticaria, Dry Skin, Lichen planus (Local/ general), Hyperpigmentation/Blackish discoloration/ Red spots of skin (Local/general), Dryness of skin (Local/ General), Vitiligo/ White patches on skin, Psoriasis, Keratosis, Full body itching, Follicular cystitis |
| **Musculoskeletal Disorders** | Difficulty in walking, CS, Muscular weakness |
| **Nutritional Disorders** | Anemia, Vit. B12 Deficiency, D3 Deficiency |
| **Others** | General body weakness/ Generalized weakness, Fatigue, Hiatus hernia, Umbilical hernia, Giddiness, HIV, Fever |
